# A Novel Minimum Spanning Tree Clustering Algorithm Based on Density Core

**DOI:** 10.1155/2022/8496265

**Published:** 2022-10-05

**Authors:** Qiang Gao, Qin-Qin Gao, Zhong-Yang Xiong, Yu-Fang Zhang, Min Zhang

**Affiliations:** Key Laboratory of Dependable Service Computing in Cyber-Physical Society, Ministry of Education, Chongqing University, Chongqing 400044, China

## Abstract

Clustering analysis is an unsupervised learning method, which has applications across many fields such as pattern recognition, machine learning, information security, and image segmentation. The density-based method, as one of the various clustering algorithms, has achieved good performance. However, it works poor in dealing with multidensity and complex-shaped datasets. Moreover, the result of this method depends heavily on the parameters we input. Thus, we propose a novel clustering algorithm (called the MST-DC) in this paper, which is based on the density core. Firstly, we employ the reverse nearest neighbors to extract core objects. Secondly, we use the minimum spanning tree algorithm to cluster the core objects. Finally, the remaining objects are assigned to the cluster to which their nearest core object belongs. The experimental results on several synthetic and real-world datasets show the superiority of the MST-DC to Kmeans, DBSCAN, DPC, DCore, SNNDPC, and LDP-MST.

## 1. Introduction

Clustering analysis, which classifies the unlabeled data into some clusters, refers to a task to discover the internal structure of data or the potential data models [1]. Since the early 1950s, quite a few clustering algorithms have been put forward [2, 3]. These algorithms can be roughly classified into four categories: partition-based clustering algorithms [4, 5], hierarchical clustering algorithms [6, 7], density-based clustering algorithms [8, 9], and graph-based clustering algorithms [10–12]. Thanks to the predominant capability of discovering clusters of different shapes and sizes along with outliers, density-based and partition-based clustering technologies are widely used in the fields of health care [13], information security [14], the Internet [15], etc. Besides, clustering is also a vital key for analyzing big data.

Partition-based clustering algorithms are the simplest and most fundamental clustering algorithms. They organize the data objects into several nonoverlapping partitions where each partition represents a cluster, and each data object belongs to one cluster [16]. Nevertheless, traditional partition-based methods usually cannot find clusters with arbitrary shapes.

However, identifying clusters with arbitrary shapes is a very important task in the applications of clustering algorithms. Since the density-based clustering algorithm does not need to know the number of clusters in the dataset in advance and can effectively process datasets with arbitrary shapes, it has always been the focus of research in clustering algorithms. The idea of the density-based clustering algorithm [17] is that the clusters in the dataset are a collection of dense data regions separated by sparse data regions. DBSCAN [18] and DPC [8] are two typical algorithms among density-based algorithms. The DBSCAN algorithm manually sets the neighborhood radius *ε* and the minimum density points *Minpts*, and it classifies data points into core points, boundary points, and outliers. Besides, DBSCAN can be effectively applied to datasets with complex shapes, and outliers can be detected during the clustering process. However, the algorithm gets poor clustering results for multidensity datasets. Furthermore, for different datasets, setting different parameters in DBSCABN will get unstable clustering results. The DPC (density peak clustering) algorithm was published in the journal Science in 2014, and it holds the idea that cluster centers are characterized by a higher density and a relatively longer distance [8]. However, the DPC still has some drawbacks. Firstly, a threshold *dc* needs to be set by users. Besides, the cluster centers are obtained by the decision graph, which has certain human factors.

To improve the performance of DPC, DPC-KNN-PCA [19] and SNN-DPC [20] have been proposed. DPC-KNN-PCA integrates PCA, DPC, and KNN to avoid the defects of DPC. However, this density-based algorithm still cannot recognize clusters containing manifold distributions [21]. To solve the defect of the use of threshold *dc*, paper [20] proposed a shared-nearest-neighbor-based clustering by the fast search and find of density peak (SNN-DPC) algorithm. The computation of its local density *ρ* and distance from the nearest larger density point *δ* takes the information of the nearest neighbors and the shared neighbors into consideration. The assignment process in DPC is sensitive and of low fault tolerance. For example, if a data point is assigned incorrectly, then the subsequent assignment will magnify the error, resulting in more errors that will have a seriously negative impact on the clustering process. Therefore, the paper [20] adopted a two-step assignment to solve the drawbacks of DPC. Yet, SNN-DPC still has several apparent defects. Firstly, the number of shared nearest neighbors *k* needs to be set through manual experience. Besides, SNN-DPC still utilizes a decision graph to select cluster centers. MST-based clustering methods [22, 23] do not assume that data points are grouped around centers or separated by regular geometric curve. Instead, they use tree edge information to divide a dataset into clusters and are able to recognize clusters with arbitrary shapes. Clustering algorithms based on the minimum spanning tree (MST) are able to detect clusters with arbitrary shapes; however, they are time-consuming and susceptible to noise points. The algorithm in [24] uses a new distance between local density peaks based on shared neighbors to construct a minimum spanning tree on the local density peaks, which excludes the interference of noise points and reduces the running time of MST-based clustering algorithms. Nevertheless, LDP-MST still needs input parameters, which means that the algorithm cannot exclude the interference of human factors.

To resolve the problem mentioned above, we propose a novel clustering algorithm (called the MST-DC). Firstly, we automatically get the reverse neighbors of each object based on the concept of natural neighbor searching. Secondly, we obtain the core objects according to the number of reverse neighbors of each object. Thirdly, based on the Prim algorithm in the graph theory, we construct a minimum spanning tree of the core objects to obtain the clustering result of core objects. Lastly, unallocated objects are marked as the label of their nearest local core objects. There is no need to set parameters in MST-DC. Furthermore, MST-DC can be applied to complex patterns with extremely large variations in density.

The remainder of this paper is organized as follows: Section 2 presents a brief overview of density core and natural neighbors; Section 3 presents the clustering algorithm (MST-DC); Section 4 presents the analysis of synthetic datasets and real datasets; and finally, Section 5 presents the summary of this paper and future work.

## 2. Related Work

We review related works on density core [25] and natural neighbors [26] in this section, which are originally mentioned by Dai et al. [25].

### 2.1. Density Core

There exist some intrinsic defects in centroid-based clustering methods, including shape loss, false distances, and false peaks, which cause centroid-based methods to fail when applied to complex patterns [27]. Thence, Chen et al. [27] proposed a hybrid decentralized approach named DCore, which was used to overcome these defects in centroid-based clustering methods. Density cores can roughly maintain the shape of the cluster and be located far from each other.

As is known to all, the mean shift algorithm can identify nonspherical patterns by shifting tracks. Thus, DCore uses mean shift and *k*-center to obtain convergence points. DCore is a hybrid method that can decentralize each density peak into a loose density core, which can refrain some intrinsic defects in centroid-based clustering approaches. The application of DCore in different datasets indicates that it can perform well in many complex datasets. Nevertheless, it still has some obvious limitations:

1. DCore algorithm uses a global fastened scanning radius to search convergent points and density cores. For a dataset with multiple density levels, it cannot obtain an ideal density representative point using a global fastened scanning radius, and thus cannot obtain ideal clustering results.
2. In order to filter noise, the DCore algorithm adopts three filtering strategies. However, it is usually difficult to determine a specific pattern, so it is difficult to select the corresponding strategy to detect outliers and noise.
3. The DCore algorithm needs to adjust five parameters to attain better clustering results. It is difficult to find the ideal combination of parameters to make the clustering perform better.

### 2.2. Natural Neighbor

Natural neighbor [26] is a new concept that originates from the reality that the number of one's real friends should be the number of how many people are taken him or her as friends, and he or she takes them as friends at the same time. For example, if object *p* regards object *q* as a neighbor and object *q* regards object *p* as a neighbor at the same time, then object *q* is one of the natural neighbors of object *p*. To put it another way, the main idea of the natural neighbor stable structure is that objects lying in sparse regions possess a small number of neighbors. In contrast, objects lying in dense regions possess a large number of neighbors [28]. Thus, the natural neighbor stable structure of objects is formulated as follows:

(1) $$\begin{array}{c} \begin{array}{c} \left(\forall p\right)\left(\exists q\right)\left(k\in N\right)\wedge \left(p\neq q\right)⟶\left(q\in KNN_{k}\left(p\right)\right)\wedge \left(p\in KNN_{k}\left(q\right)\right) \end{array}\text{,} \end{array}$$

where *KNN*_*k*_(*p*) is the *k* nearest neighbor of object *p*.

**Definition 1 .**

(k nearest neighbors). The *k* nearest neighbors of object *p* are a set of objects *q* in dataset *X* with dist(*p*, *q*) ≤ dist(*p*, o), that is,

(2) $$\begin{array}{c} KNN_{k}\left(p\right)=\left\{q\left|\forall q\in X,\text{dist}\left(p,q\right)\leq \text{dist}\left(p,o\right)\right.\right\}\text{,} \end{array}$$

where dist (*p*, *o*) is the distance between the object *p* and the *k*th object *o*.

**Definition 2 .**

(Reverse neighbors). The reverse neighbors of object *p* are a set of objects *q* that regard *p* as its *k* nearest neighbor, i.e., *RNN*_*k*_(*p*)={*q* ∈ *X*|*p* ∈ *KNN*_*k*_(*q*)}.

The natural neighbor stable structure's formation process is represented as follows: continuously expand the neighbor searching range *k* increasing from 1 to *λ* (*λ* is named natural neighbor eigenvalue (NaNE)) [26]; in each searching process, calculate the number of reverse neighbors of each object and judge the following two conditions: (1) all objects have reverse neighbors and (2) the number of objects without reverse neighbors remains unchanged; and when one of these conditions is met, the natural neighbor stable structure is formed. The searching range *k* is equal to the natural characteristic value *λ* at this moment. Therefore, *λ* is obtained by

(3) $$\begin{array}{c} \lambda =\min\;\left\{\left.k\right|\overset{n}{\underset{p=1}{\sum }}f\left(nb_{k}\left(p\right)\right)=0\;\text{or }\overset{n}{\underset{p=1}{\sum }}f\left(nb_{k}\left(p\right)\right)=\overset{n}{\underset{p=1}{\sum }}f\left(nb_{k-1}\left(p\right)\right)\right\}\text{,} \end{array}$$

where *k* is initialized with 1, *nb*_*k*_(*p*) is the number of object *p*'s reverse neighbor in the *kth* iteration (note that *nb*_*k*_(*p*) ≥ 0), and *f*(*x*) is defined as follows:

(4) $$\begin{array}{c} f\left(x\right)=\left\{\begin{array}{cc} 0\text{,} & \text{otherwise},\\ 1\text{,} & \text{if}\;x==0\text{.} \end{array}\right. \end{array}$$

Based on the above concepts, the natural neighbor is defined as follows:

**Definition 3 .**

(Natural neighbors). For each object *p*, the *k* nearest neighbors are natural neighbors, denoted as *NaN*(*p*), where *k* is equal to the natural characteristic value *λ*.

Apparently, each object has the same number of natural neighbors in this paper. The details of the natural neighbor searching algorithm are shown in Algorithm 1.

## 3. The Proposed Algorithm

Based on the idea of density core, we apply the reverse nearest neighbors in the natural neighbor searching algorithm to extract density core sets. And density core sets can maintain the general shape of clusters well. Subsequently, we construct a minimum spanning tree of density core points for clustering. Besides, we use the reverse nearest neighbors in the process of natural neighbor searching, which does not require any parameter settings. MST-DC can recognize extremely complicated clusters with large variations in density.

### 3.1. Density Core Set

According to Algorithm 1, we calculate the number of reverse nearest neighbors for each object. Since the number of core objects' reverse nearest neighbors are greater than that of noncore objects' reverse nearest neighbors, we use the number of reverse nearest neighbors to extract core objects. The definition of the core object is as follows:

**Definition 4 .**

(Core object). If one object can be considered as a core object, it must satisfy the following formula:

(5) $$\begin{array}{c} R\text{Core}=\left\{p\left|\forall p\in SRNN\left(p\right)\geq k\right.\right\}\text{,} \end{array}$$

where SRNN(*p*) represents the number of reverse nearest neighbors of object *p*, and *k* represents the natural characteristic value.

**Figure 1:**
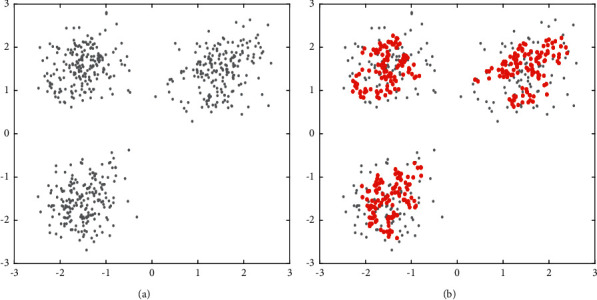
Density core points extracted based on a reverse nearest neighbor number.

As is mentioned above, each data point regards its neighbor point as a potential density core point. This neighbor point is a true density core point when there are enough data objects to treat it as a potential density core point.  Figure 1(a) shows an original dataset with three clusters. After the core point extraction process, as shown in Figure 1(b), the red regions represent potential clusters, and each point in the red regions is a core object. The gray points are noncore objects. Algorithm 2 presents the process of finding core sets, which can roughly retain the shape of clusters.

### 3.2. Clustering Core Objects

After we obtain the density core points, how to cluster the density core points becomes a key task. We propose a method of clustering density core points based on the minimum spanning tree. The density core sets extracted in the dataset retain the general shape of the cluster, while the distance between the density core sets is further apart. After constructing the minimum spanning tree, it is easier to find the longest edge of the tree for cutting.

The process of clustering the density core points based on the minimum spanning tree is as follows: Firstly, we construct a minimum spanning tree based on the set of density core points, and secondly, we cut off the edges whose length is greater than the trimming threshold. Afterwards, we can obtain the cluster of the density core sets according to tree structure information after trimming. The trimming threshold cut*θ* is defined as follows:where mean(E dg e) represents the average value of all the edge weights in the minimum spanning tree, and st d(E dg e) represents the standard deviation of all the edge weights in the minimum spanning tree. *ω* is an empirical value, and its value range is [2, 5], which has been verified by a large number of experiments. When *ω* = 3, it meets the requirements of most datasets. In this article, we choose *ω* = 3 as the experimental parameter. The setting of the trimming threshold is based on statistical principles, which can check whether there are outliers in the data. The length of the edge of the minimum spanning tree constructed conforms to the Gaussian distribution, and the edge we need to trim is the longer one located between different clusters.

**Definition 5 .**

(Trimming threshold). The threshold is derived from the overall minimum spanning tree.

(6) $$\begin{array}{c} cut\theta =mean\left(Edge\right)+\omega *st\;d\left(Edge\right)\text{,} \end{array}$$

**Figure 2:**
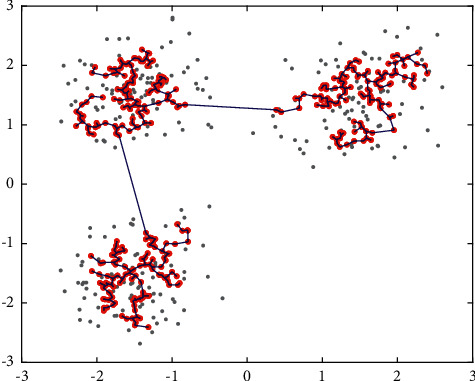
Minimum spanning tree based on density core points.

**Figure 3:**
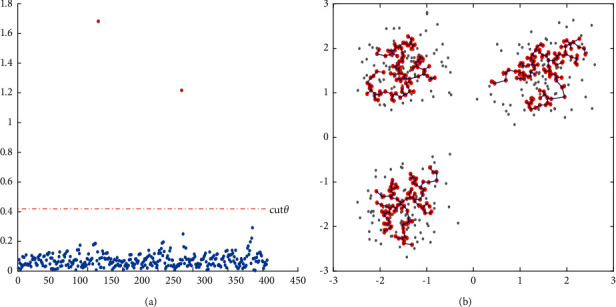
Obtaining each subcluster based on cut*θ*.

The steps of clustering density core points are as follows:

1. We utilize the Prim algorithm to construct the minimum spanning tree on all the density core points. The length of the edge, which is computed based on the Euclidean distance, is used as the weight in the minimum spanning tree. The minimum spanning tree we build based on the density core points is shown in Figure 2.
2. After building the minimum spanning tree, we get a minimum spanning tree edge set. Since we build the minimum spanning tree based on the density core, the weight of the tree in the same cluster is relatively small, and the change of weight is small as well. The weight of the edge between different clusters is larger, so it is easier for us to find the edges connecting different clusters and cut them off. As shown in Figure 3(a), the colored dots represent the weights of the edges of the minimum spanning tree. The weights of the two edges (red dots) are much larger than the weights of the other edges (blue dots). The red dotted line indicates the trimming threshold we calculated, and the trimming threshold can effectively identify the longer sides in the minimum spanning tree; Figure 3(b) shows the minimum spanning tree of the subcluster after cutting off the edge greater than the trimming threshold. From the figure, we can see that the red density core points have been divided into three parts, and each part of the density core has a minimum spanning tree after cutting.
3. We cluster the density cores according to the minimum spanning subtree structure retained by the density core points of each cluster after trimming, namely, assigning the points on the same minimum spanning subtree to the same cluster.

According to the description of the above steps, the specific steps of clustering density core points are shown in Algorithm 3.

### 3.3. The MST-DC Clustering Algorithm

In this section, we introduce a novel clustering algorithm, namely, MST-DC. The basic steps are as follows: firstly, we find the reverse nearest neighbors of each object according to the natural neighbor searching algorithm; secondly, we use formula (5) to get the core objects; thirdly, we build the minimum spanning tree of the density core set *R*Core, and the edge between the clusters is cut according to formula (6), and then, the density cores are clustered according to the obtained subcluster tree; fourthly, we apply the concept of outlier clusters in paper [29] to eliminate erroneous clusters. In this paper, a novel outlier cluster detection algorithm called ROCF is proposed based on the concept of mutual neighbor graph and on the idea that the size of outlier clusters is usually much smaller than the normal clusters; and finally, the noncore points are assigned to the clusters to which their closest density core points belong. The overall steps of the MST-DC algorithm are shown in Algorithm 4.

### 3.4. The Complexity Analysis

Based on the description of the MST-DC clustering algorithm in the previous sections, the time complexity of MST-DC depends on following parts: (1) we use the natural neighbor algorithm optimized by *KD* − tree [30] to obtain the reverse nearest neighbors of each data point, the natural eigenvalue, and the Euclidean distance of the data points, and its time complexity is *O*(*n* log(*n*)); (2) the process of extracting core points is equivalent to traversing data points, and its time complexity is *O*(*n*); (3) the time complexity of clustering the core points based on the minimum spanning tree is mainly focused on the Prim algorithm to establish the minimum spanning tree. This paper uses the heap-optimized Prim algorithm, whose time complexity is *O*(*m* log(*m*)), where *m*(*m* ≪ *n*) represents the number of obtained core points; and (4) we assign the remaining points to their nearest density core with time complexity of *O*(*l*), where *l*(*l* < *n*, *n*=*l*+*m*) represents the number of remaining noncore points. In summary, the total time complexity of MST-DC is *O*(*n* log(*n*)).

## 4. Experiments and Analysis

### 4.1. Experimental Design and Environment

In this section, we demonstrate the effectiveness of the proposed clustering algorithm, respectively, on some artificial datasets and UCI real datasets. And we compare the MST-DC with well-known and state-of-the-art clustering methods, including Kmeans [5], DBSCAN [18], DPC [8], DCore [27], SNN-DPC [20], and LDP-MST [24].

The experiment is conducted on a MATLAB 2018a computer equipped with Intel Core 2.20 GHz CPU, 8 GB RAM, and Windows 10 operating system. The MATLAB code is available from https://github.com/qczggaoqiang/MST-DC

### 4.2. Metrics for Measurement

We adopt Accuracy [31], *F* − Measure [32], and NMI (normalized mutual information) [33] to test the performance of our proposed algorithm MST-DC and six comparison algorithms. The upper limit of the three indexes is 1. The larger the values of the three indexes are, the better the clustering result is.

We chose Accuracy as the first evaluation indicator. For *n* objects *x*_*i*_ ∈ *D*^*j*^, *p*_*i*_ and *c*_*i*_ are the intrinsic category label and the predicted cluster label of *x*_*i*_, respectively, and the calculation formula of Accuracy is as follows:

(7) $$\begin{array}{c} \text{Accuracy}=\overset{n}{\underset{i=1}{\sum }}\frac{\delta \left(p_{i},\text{map}\left(c_{i}\right)\right)}{n}\text{,} \end{array}$$

where map(.) is a mapping function that maps the predicted cluster label and its intrinsic cluster label by the Hungarian algorithm [34]; let *δ*(*a*, *b*) equal to 1 if *a*=*b* or equal to 0 otherwise. Accuracy ∈ [0,1]; namely, the higher the values of the Accuracy are, the better the clustering performance will be.

*F* − Measure includes both the precision *P* and the recall *R*. *P* is the ratio between the number of correct positive results and the number of all positive results returned by the classifier. *R* is the ratio between the number of correct positive results and the number of all samples that should have been identified as positive *P*, *R*, and *F* − Measure, which are defined by formulas (8)–(10). *M*_*j*_ is a set of the number of all samples that should have been identified as positive. *C*_*i*_ is a set of the number of all positive results returned by the classifier.

(8) $$\begin{array}{c} P\left(M_{j},C_{i}\right)=\frac{\left|M_{j}\cap C_{i}\right|}{\left|C_{i}\right|}\text{,} \end{array}$$

(9) $$\begin{array}{c} R\left(M_{j},C_{i}\right)=\frac{\left|M_{j}\cap C_{i}\right|}{\left|M_{j}\right|}\text{,} \end{array}$$

(10) $$\begin{array}{c} F\left(M_{j},C_{i}\right)=\frac{2\times P\left(M_{j},C_{i}\right)\times R\left(M_{j},C_{i}\right)}{P\left(M_{j},C_{i}\right)+R\left(M_{j},C_{i}\right)}\text{.} \end{array}$$

The mutual information (*MI*) [32] can be used to measure the information shared by two clusters, given a set *S* of *N* data points, and two partitions of set *S*, namely, *X*={*X*_1_, *X*_2_,…, *X*_*r*_}, and *Y*={*Y*_1_, *Y*_2_,…, *Y*_*s*_}. Suppose that we select a point at random from *S*, then the probability that the point belongs to cluster *X*_*i*_ is

(11) $$\begin{array}{c} P\left(i\right)=\frac{\left|X_{i}\right|}{N}\text{.} \end{array}$$

Entropy can be described as the information conveyed by the uncertainty that a randomly selected point belongs to a certain cluster. The entropy of the cluster *X* is given by the following formula:

(12) $$\begin{array}{c} H\left(X\right)=-\overset{r}{\underset{i=1}{\sum }}P\left(i\right)\times \log\;P\left(i\right)\text{.} \end{array}$$

The *MI* between the clusters *X* and *Y* is defined by

(13) $$\begin{array}{c} MI\left(X,Y\right)=\overset{r}{\underset{i=1}{\sum }}\overset{s}{\underset{j=1}{\sum }}P\left(i,j\right)\times \log\frac{P\left(i,j\right)}{P\left(i\right)P\left(j\right)}\text{.} \end{array}$$

The *NMI* is calculated as follows:

(14) $$\begin{array}{c} NMI\left(X,Y\right)=\frac{2\times MI\left(X,Y\right)}{H\left(X\right)+H\left(Y\right)}\text{.} \end{array}$$

### 4.3. Experiments on Synthetic Datasets

**Figure 4:**
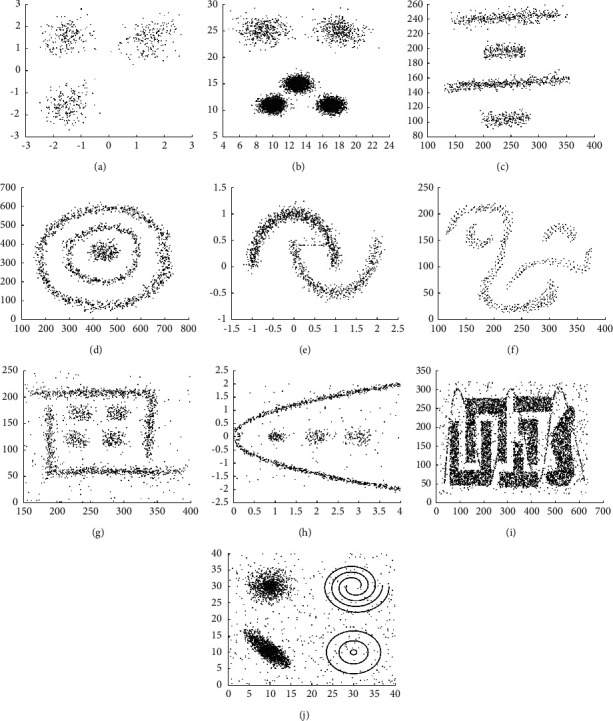
Ten original synthetic datasets. (a) D1, (b) D2, (c) D3, (d) D4, (e) D5, (f) D6, (g) D7, (h) D8, (i) D9, and (j) D10.

We first conduct comparison experiments based on ten synthetic datasets. As shown in Table 1, the characteristics of ten synthetic datasets are described. Moreover, ten original synthetic datasets are displayed in Figure 4. D1 and D2 contain spherical clusters with a different number of clusters and densities. D1 contains three clusters and a total of 600 objects. D2 consists of five clusters with skewed distribution, and a total of 6699 objects. In contrast, the remaining synthetic datasets contain clusters with arbitrary shapes. D3 is composed of four-line clusters and a total of 1268 objects. D4 has a spherical cluster in the middle of two ring clusters and a total of 1897 objects. D5 is composed of two moon manifold clusters with 1532 objects, including noise objects. D6 includes four manifold clusters and a total of 630 objects. D7 has four spherical clusters in two right-angle line clusters with some noise objects, and a total of 1916 objects. D8 has three spherical clusters in one manifold cluster with several noise objects, and a total of 1427 objects. D9 is composed of six square clusters that cross and parallel with each other, and a total of 8000 data objects including some noise objects. D10 consists of three circle clusters, two spiral clusters, and two spherical clusters with a total of 8533 objects, including some noise objects.

The parameter settings of each clustering algorithm in the ten synthetic datasets are displayed in Table 2. As for the Kmeans algorithm, *N* represents the number of clusters in the dataset, and the initial clustering center is randomly selected. DBSCAN needs two parameters: Eps and Minpts. The cutoff distance *dc* of DPC is set as 2%. SNNDPC needs the parameter *K* to find *k* nearest neighbors. We test different *K* to achieve better results. The results of DCore are affected by the selection of parameters *r*1, *r*2, *T*_1_, *T*_*n*_, and *R*. We use different parameter settings to achieve better results. The LDP-MST algorithm needs to set the parameter N manually. Concerning MST-DC, there is no need to set parameters. In Table 2, the symbol “ — ” indicates that there is no need to set parameters in MST-DC.

**Figure 5:**
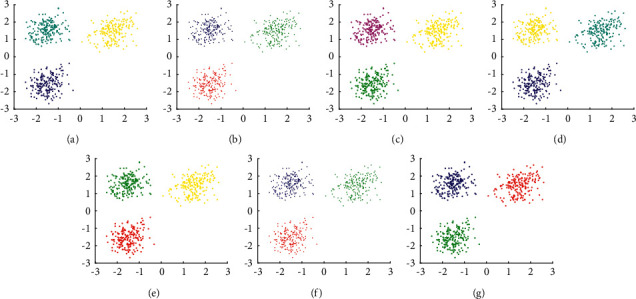
Results of 7 clustering algorithms in D1. (a) Kmeans, (b) DBSCAN, (c) DPC, (d) DCore, (e) SNNDPC, (f) LDP-MST, and (g) MST-DC.

The experimental result of D1 is shown in Figure 5. It shows that all clustering algorithms can find correct clusters in D1, which means that these algorithms are effective for uniformly distributed spherical datasets. However, except MST-DC, other algorithms need parameter settings.

**Figure 6:**
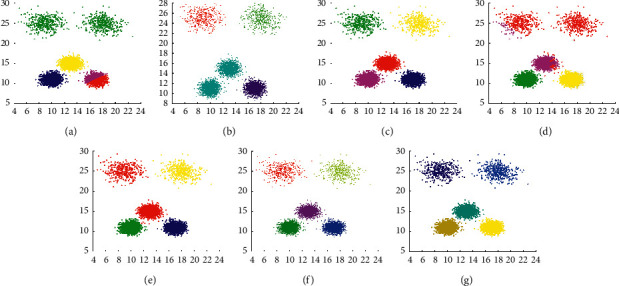
Results of 7 clustering algorithms in D2. (a) Kmeans, (b) DBSCAN, (c) DPC, (d) DCore, (e) SNNDPC, (f) LDP-MST, and (g) MST-DC.

Figure 6 shows that DPC, LDP-MST, and MST-DC can get the correct clustering on D2, while Kmeans, DBSCAN, DCore, and SNNDPC cannot. The number of clusters in the Kmeans algorithm is input by users. Because it cannot recognize clusters with different densities, the low-density area is mistakenly recognized as the same cluster, while the high-density area is erroneously partitioned as well. Because the choice of Eps is too large, DBSCAN aggregates D2 into four clusters. Because of the improper choices of parameters, DCore and SNNDPC cannot correctly identify clusters in the D2 dataset. Therefore, Figure 6 displays that using global fixed parameter settings for multidensity patterns is not applicable.

**Figure 7:**
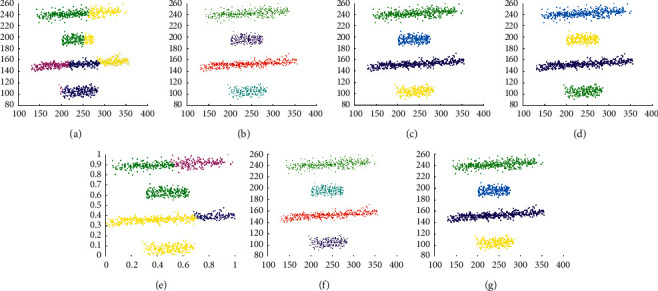
Results of 7 clustering algorithms in D3. (a) Kmeans, (b) DBSCAN, (c) DPC, (d) DCore, (e) SNNDPC, (f) LDP-MST, and (g) MST-DC.

The experimental results on D3 are shown in Figure 7. It shows that except Kmeans and SNNDPC, other algorithms can well find the correct clusters. It also illustrates that Kmeans cannot be applied to line cluster datasets. Moreover, the incorrect setting of parameters leads to incorrect clustering results.

**Figure 8:**
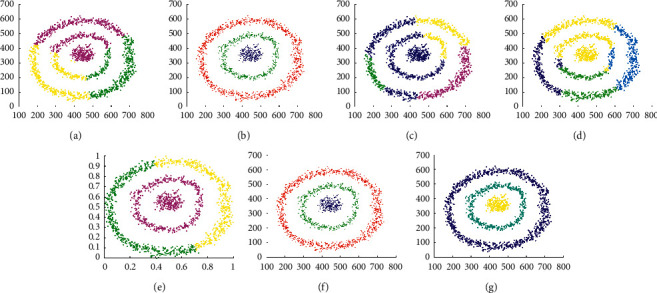
Results of 7 clustering algorithms in D4. (a) Kmeans, (b) DBSCAN, (c) DPC, (d) DCore, (e) SNNDPC, (f) LDP-MST, and (g) MST-DC.

The experimental results on D4 are shown in Figure 8. It demonstrates whether those algorithms can process circle clusters or not. It shows that Kmeans, DPC, DCore, and SNNDPC algorithms are not suitable for datasets containing circular clusters, and the correct clustering results cannot be obtained. DBSCAN, LDP-MST, and MST-DC algorithms can find the correct clusters for D4.

**Figure 9:**
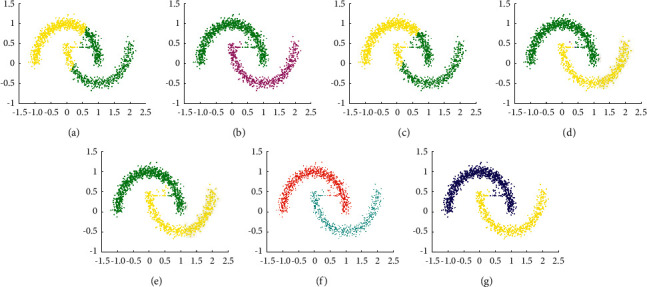
Results of 7 clustering algorithms in D5. (a) Kmeans, (b) DBSCAN, (c) DPC, (d) DCore, (e) SNNDPC, (f) LDP-MST, and (g) MST-DC.

**Figure 10:**
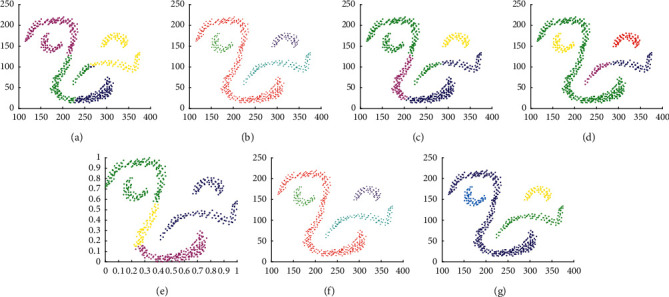
Results of 7 clustering algorithms in D6. (a) Kmeans, (b) DBSCAN, (c) DPC, (d) DCore, (e) SNNDPC, (f) LDP-MST, and (g) MST-DC.

**Figure 11:**
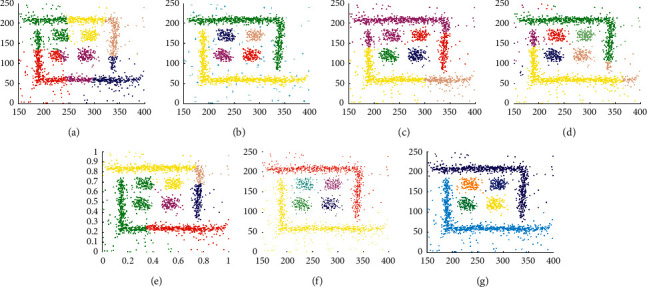
Results of 7 clustering algorithms in D7. (a) Kmeans, (b) DBSCAN, (c) DPC, (d) DCore, (e) SNNDPC, (f) LDP-MST, and (g) MST-DC.

The clustering results displayed in Figures 9–11 demonstrate whether those algorithms can process clusters with the arbitrary shape or not. The results of DBSCAN, LDP-MST, and MST-DC are similar. Three of them can find the clusters of the three datasets correctly. As shown in Figure 9(d), DCore mistakenly identifies only two points in D5. Besides, none of Kmeans, DPC, DCore, and SNNDPC can find the right clusters of D5, D6, and D7 datasets.

**Figure 12:**
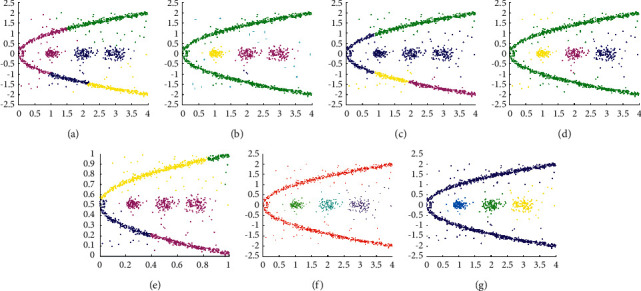
Results of 7 clustering algorithms in D8. (a) Kmeans, (b) DBSCAN, (c) DPC, (d) DCore, (e) SNNDPC, (f) LDP-MST, and (g) MST-DC.

The clustering results shown in Figure 12 display that MST-DC, LDP-MST, and DCore have recognized the D8 datasets correctly, while Kmeans, DBSCAN, DPC, and SNNDPC have not. In other words, MST-DC, LDP-MST, and DCore can be good at dealing with manifold structure datasets. In Figure 12(b), DBSCAN can detect the manifold cluster. Yet, it does not work for the datasets combined with spherical clusters and manifold cluster.

**Figure 13:**
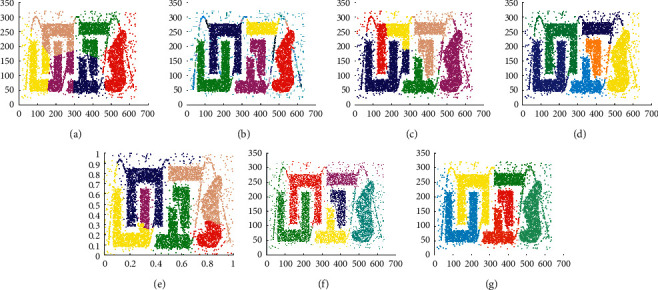
Results of 7 clustering algorithms in D9. (a) Kmeans, (b) DBSCAN, (c) DPC, (d) DCore, (e) SNNDPC, (f) LDP-MST, and (g) MST-DC.

**Figure 14:**
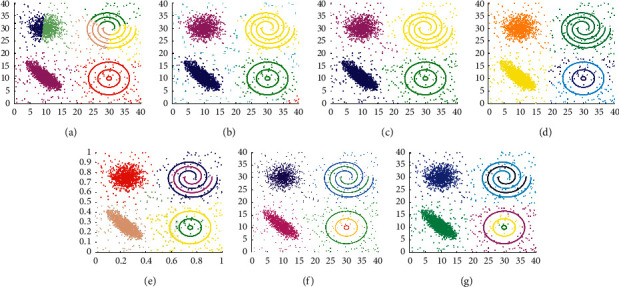
Results of 7 clustering algorithms in D10. (a) Kmeans, (b) DBSCAN, (c) DPC, (d) DCore, (e) SNNDPC, (f) LDP-MST, and (g) MST-DC.

The experimental results displayed in Figures 13 and 14 are used to evaluate the clustering performance on D9 and D10, which are more complex patterns. As shown in Figure 13, only DCore, LDP-MST, and MST-DC can detect rectangle clusters. Because of inappropriate parameter settings, DBSCAN and SNNDPC did not get impressive clustering results, and neither did Kmeans and DPC. The clustering results shown in Figure 14 demonstrate that only the LDP-MST and MST-DC algorithms obtained the correct clusters. DBSCAN and DPC have similar results that both of them cannot deal with spiral clusters and circle clusters. Although SNNDPC can detect the spiral clusters, they fail to handle the circle clusters. DCore can detect circle clusters; however, it cannot detect the spiral clusters. Thence, MST-DC can be applied to more complex situations without parameter settings.

From Figures 5–14, we can see that MST-DC performs better than other algorithms. Moreover, there is no need to set any parameters so that several intrinsic flaws of other algorithms can be avoided.

The running time of seven clustering algorithms on the synthetic datasets is shown in Table 3. It is obvious that although MST-DC runs slower than Kmeans, DBSCAN, and LDP-MST, it runs faster than DPC and SNNDPC evidently. What is more, the consuming time of MST-DC is similar to that of DCore.

### 4.4. Experiments on Real-World Datasets

To further prove the superiority of the MST-DC algorithm, we also apply the proposed method to six real-world datasets, including Segmentation, Pageblock, Iris, Control, Column2C, and Breast, obtained from the University of California, Irvine (UCI) Machine Learning Repository. The characteristics of six real datasets are displayed in Table 4.  Table 5 illustrates the parameter setting of each clustering algorithm in six real-world datasets of UCI, and the symbol “ — ” indicates that there is no need to set parameters in MST-DC.  Table 6 shows the clustering performance of seven clustering algorithms according to three external criteria, namely, Accuracy, *F* − Measure, and NMI.  Table 7 shows the running time of each algorithm, where “ — ” indicates that the algorithm has not run within the specified time (20 minutes).

As shown in Table 6, except for the Iris dataset, the clustering performance of the MST-DC algorithm is superior to the Kmeans, DBSCAN, DPC, DCore, SNNDPC, and LDP-MST algorithms on other datasets. The LDP-MST algorithm obtains the optimal clustering results on the Iris dataset in terms of *Accuracy*, NMI, and *F* − Measure. Besides, LDP-MST achieves the best results on Segmentation and Control datasets in terms of Accuracy, and on Column2C dataset in terms of NMI. On relatively simple datasets, the Accuracy, NMI, and *F* − Measure values of the Kmeans, DBSCAN, DPC, and SNNDPC algorithms are high; however, on datasets with higher dimensions or complex structures, the four algorithms have poor performances. Except for the Segmentation dataset, the DCore algorithm can obtain a relatively good clustering effect on multiple datasets. However, the DCore algorithm has a major flaw that it needs to set five parameters manually. It is usually very difficult to adjust the parameter combination for better clustering results.

According to Table 7, MST-DC is slower than the Kmeans, DBSCAN, and LDP-MST algorithms on the UCI dataset. However, the MST-DC algorithm is much faster than the DPC and SNNDPC algorithms. The running time of the MST-DC and DCore algorithms is similar on most datasets.

From the above analysis, we conclude that MST-DC provides an overall good performance in clustering compared with the other existing methods. Firstly, the MST-DC algorithm employs a natural neighbor algorithm to obtain the reverse neighbor information and then extracts the density core point according to the number of reverse neighbors and natural characteristic value. This process does not need to set parameters, while other algorithms need to set parameters manually. Secondly, MST-DC only utilizes the density core points instead of all points to build the minimum spanning tree, which reduces the computational cost while excluding the interference of noise points. Thirdly, the MST-DC algorithm can recognize complex datasets efficiently and accurately. However, compared with Kmeans, DBSCAN, and LDP-MST, the time efficiency of the MST-DC algorithm is not optimal, which is worth further exploration.

## 5. Conclusions

In this paper, we propose a novel clustering algorithm named MST-DC. The process of the algorithm has the following four steps: firstly, we automatically obtain the reverse neighbors of each object based on the concept of natural neighbor searching without any parameters set by the user; secondly, we obtain the core objects according to the number of reverse neighbors of each object; thirdly, we construct a minimum spanning tree of the core objects to obtain the clustering result of core objects; and finally, unallocated objects are marked as the label of their nearest local core objects.

The experimental results of synthetic and real-world datasets demonstrate that MST-DC can detect quite complex patterns with large variations in density. Besides, unlike most clustering methods, there is no need to set parameters in MST-DC. The concept of natural neighbor can automatically obtain the only parameter *k* used by MST-DC. Therefore, our proposed algorithm, MST-DC, is superior to other algorithms.

Nevertheless, there are several aspects to be improved in this paper. Firstly, the similarity measure based on the Euclidean distance is used when acquiring natural neighbor information, extracting density cores, and assigning remaining points; however, the Euclidean distance is prone to Dimensional Curse in high-dimensional data spaces, resulting in poor clustering effects. Therefore, we will explore the adaptability of this algorithm to high-dimensional data in the future. Secondly, the approach to assign the remaining points in this paper is to directly assign them to the cluster where the closest density core is located. Subsequently, we will study the new method of allocating the remaining points.

## Figures and Tables

**Algorithm 1:**
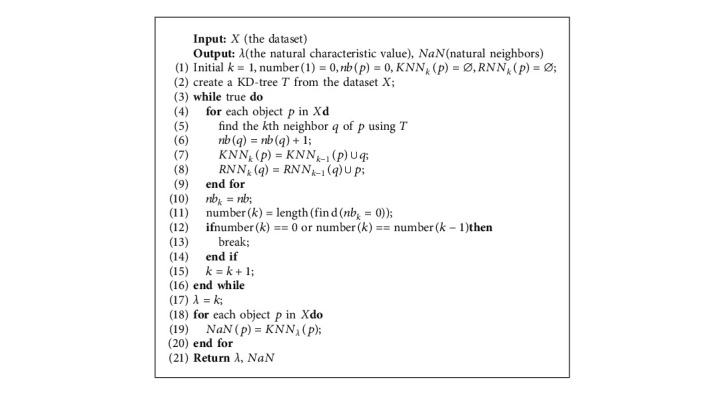
Natural neighbor searching algorithm (NaN-searching).

**Algorithm 2:**
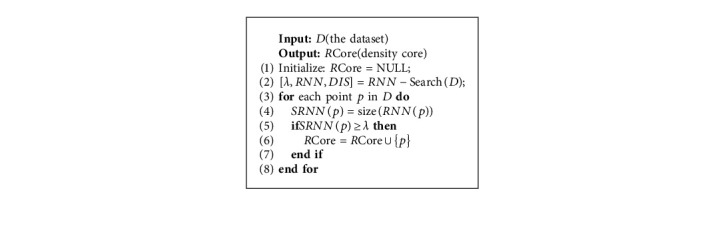
Find core set (find-cores).

**Algorithm 3:**
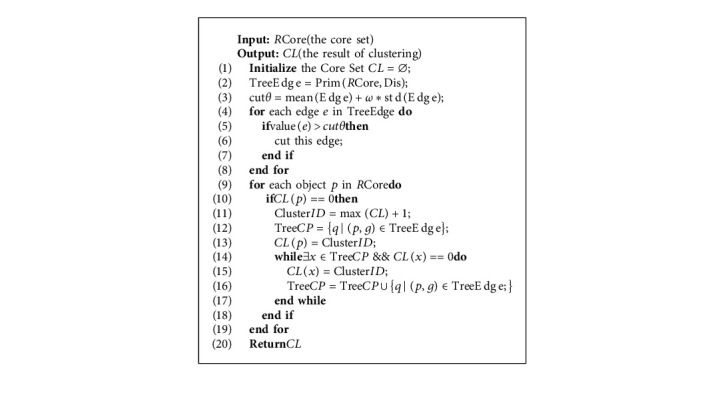
Clustering core object.

**Algorithm 4:**
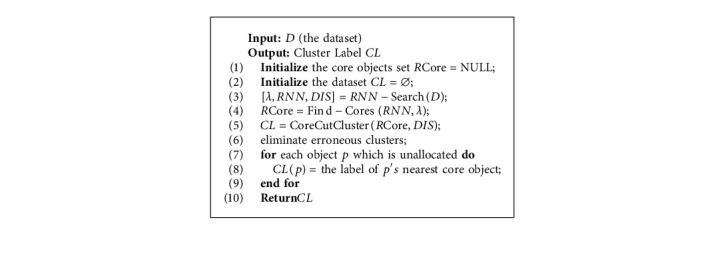
MST-DC.

**Table 1:** Characteristics of synthetic datasets.

Datasets	Number of instances	Number of attributes	Number of clusters
D1	600	2	3
D2	6699	2	5
D3	1268	2	4
D4	1897	2	3
D5	1532	2	2
D6	630	2	4
D7	1916	2	6
D8	1427	2	4
D9	8000	2	6
D10	8533	2	7

**Table 2:** Parameter settings of each clustering method in the ten synthetic datasets.

Datasets	Kmeans	DBSCAN	DPC (%)	DCore	SNNDPC	LDP-MST	MST-DC
D1	*N* = 3	Eps = 0.5 Minpts = 5	dc = 2	*r*1 = 0.5; *r*2 = 0.25; *R* = 0.6; *T*1 = 30; *Tn* = 4	*K* = 5	*N* = 3	−

D2	*N* = 5	Eps = 0.8 Minpts = 5	dc = 2	*r*1 = 0.5; *r*2 = 0.45; *R* = 0.5; *T*1 = 50; *Tn* = 15	*K* = 4	*N* = 5	−

D3	*N* = 4	Eps = 10 Minpts = 5	dc = 2	*r*1 = 14; *r*2 = 13; *R* = 15; *T*1 = 40; *Tn* = 8	*K* = 10	*N* = 4	−

D4	*N* = 3	Eps = 20 Minpts = 5	dc = 2	*r*1 = 20; *r*2 = 18; *R* = 20; *T*1 = 30; *Tn* = 8	*K* = 10	*N* = 3	−

D5	*N* = 2	Eps = 0.1 Minpts = 8	dc = 2	*r*1 = 0.3; *r*2 = 0.1; *R* = 0.2; *T*1 = 20; *Tn* = 5	*K* = 5	*N* = 2	−

D6	*N* = 4	Eps = 15 Minpts = 5	dc = 2	*r*1 = 20; *r*2 = 18; *R* = 21; *T*1 = 10; *Tn* = 6	*K* = 10	*N* = 4	−

D7	*N* = 6	Eps = 6 Minpts = 5	dc = 2	*r*1 = 14; *r*2 = 13; *R* = 14; *T*1 = 40; *Tn* = 8	*K* = 5	*N* = 6	−

D8	*N* = 4	Eps = 0.25 Minpts = 4	dc = 2	*r*1 = 0.4; *r*2 = 0.35; *R* = 0.5; *T*1 = 10; *Tn* = 5	*K* = 10	*N* = 4	−

D9	*N* = 6	Eps = 6 Minpts = 5	dc = 2	*r*1 = 15; *r*2 = 15; *R* = 15; *T*1 = 35; *Tn* = 15	*K* = 15	*N* = 6	−

D10	*N* = 7	Eps = 2 Minpts = 10	dc = 2	*r*1 = 1; *r*2 = 0.9; *R* = 2; *T*1 = 30; *Tn* = 10	*K* = 15	*N* = 7	−

**Table 3:** Consuming time of seven clustering methods on synthetic datasets.

Datasets	Kmeans	DBSCAN	DPC	DCore	SNNDPC	LDP-MST	MST-DC
D1	0.0041	0.0124	2.5567	0.1606	1.5501	0.0322	0.1264
D2	0.0161	0.6175	260.2096	5.1929	50.0567	0.7636	4.6564
D3	0.0129	0.0279	6.0232	0.2155	4.0169	0.0863	0.2552
D4	0.0159	0.0394	9.5406	0.4328	5.0190	0.1259	0.3316
D5	0.0731	0.0662	4.8771	0.4148	2.9375	0.1018	1.1598
D6	0.0105	0.0114	3.6684	0.1330	2.5739	0.0738	0.0841
D7	0.0136	0.0507	9.6971	0.4514	6.6409	0.1084	0.6224
D8	0.0088	0.0277	4.9969	0.3256	3.8173	0.0721	0.4609
D9	0.0613	1.0585	563.7926	9.9512	88.2114	1.8526	9.5622
D10	0.0871	0.7014	685.8524	13.5350	93.7407	1.9508	9.5915

**Table 4:** Characteristics of UCI datasets.

Datasets	Samples	Attributes		Categories
Segmentation	2310	19	7	[35]
Pageblock	5473	10	5	[35]
Iris	150	4	3	[35]
Control	600	60	6	[35]
Column2C	310	6	2	[35]
Breast	699	10	2	[35]

**Table 5:** Parameter setting of each clustering algorithm in six real-world datasets.

Datasets	Kmeans	DBSCAN	DPC (%)	DCore	SNNDPC	LDP-MST	MST-DC
Segmentation	*N* = 7	Eps = 10 Minpts = 4	dc = 2	*r*1 = 150; *r*2 = 140; *R* = 150; *T*1 = 30; *Tn* = 10	*K* = 15	*N* = 7	−

Pageblock	*N* = 5	Eps = 1 Minpts = 4	dc = 2	*r*1 = 0.2; *r*2 = 0.15; *R* = 0.2; *T*1 = 10; *Tn* = 2	*K* = 10	*N* = 5	−

Iris	*N* = 3	Eps = 1 Minpts = 4	dc = 2	*r*1 = 0.3; *r*2 = 0.2; *R* = 0.15; *T*1 = 5; *Tn* = 4	*K* = 10	*N* = 3	−

Control	*N* = 6	Eps = 1 Minpts = 4	dc = 2	*r*1 = 15; *r*2 = 13; *R* = 16; *T*1 = 10; *Tn* = 2	*K* = 8	*N* = 6	−

Column2C	*N* = 2	Eps = 11 Minpts = 5	dc = 2	*r*1 = 13; *r*2 = 12; *R* = 16; *T*1 = 20; *Tn* = 15	*K* = 3	*N* = 2	−

Breast	*N* = 2	Eps = 1 Minpts = 4	dc = 2	*r*1 = 0.4; *r*2 = 0.3; *R* = 0.15; *T*1 = 2; *Tn* = 0	*k* = 7	*N* = 2	−

**Table 6:** Comparison of accuracy, *F*-measure, and NMI between six real UCI datasets.

Datasets		Kmeans	DBSCAN	DPC	DCore	SNNDPC	LDP-MST	MST-DC
Segmentation	Accuracy	0.5117	0.5610	0.4883	0.2844	0.1429	**0.6147**	0.6134
	*F*-measure	0.5422	0.2983	0.5499	0.3866	0.2500	0.6469	**0.6782**
	NMI	0.4868	0.4029	0.5206	0.3383	0.2816	0.6870	**0.7044**

Pageblock	Accuracy	0.8993	0.9024	0.8983	0.8980	0.8983	0.8977	**0.9077**
	F-measure	0.8136	0.8608	0.8579	0.8600	0.8579	0.8091	**0.9461**
	NMI	0.0550	0.0797	0.0328	0.0355	0.0327	0.0341	**0.2010**

Iris	Accuracy	0.6667	0.6667	0.6667	0.6667	0.6600	**0.9730**	0.6667
	F-measure	0.6884	0.7778	0.7778	0.6061	0.7748	**0.9989**	0.7778
	NMI	0.5837	0.7337	0.7337	0.5263	0.7175	**0.9010**	0.7337

Control	Accuracy	0.5717	0.3367	0.5000	0.6250	0.5017	**0.6780**	0.6667
	*F*-measure	0.6765	0.3833	0.6546	0.6536	0.5387	0.6833	**0.7093**
	NMI	0.7078	0.3369	0.7198	0.6731	0.6528	0.7000	**0.7928**

Column2C	Accuracy	0.6774	0.6774	0.6774	0.7323	0.6774	0.7548	**0.7581**
	*F*-measure	0.6744	0.7361	0.7033	0.7407	0.7033	0.7623	**0.8077**
	NMI	0.1333	0.1616	0.0039	0.2262	0.0039	**0.3033**	0.2442

Breast	Accuracy	0.9571	0.8312	0.8269	0.9212	0.9413	0.9547	**0.9614**
	*F*-measure	0.9569	0.8306	0.8085	0.9511	0.5670	0.9532	**0.9612**
	NMI	0.7295	0.4642	0.3863	0.6323	0.3554	0.6868	**0.7501**

**Table 7:** Running time of 7 clustering algorithms on six real UCI datasets (unit: second).

Datasets	Kmeans	DBSCAN	DPC	DCore	SNNDPC	LDP-MST	MST-DC
Segmentation	0.0279	0.062	435.2143	0.8178	7.6259	0.2560	0.6584
Pageblock	0.0359	0.0350	—	3.2012	34.7688	0.4715	3.2736
Iris	0.004	0.0015	3.0986	0.0315	2.2313	0.0134	0.0156
Control	0.0107	0.0062	15.041	0.3087	3.0757	0.0851	0.0869
Column2C	0.0042	0.0021	15.041	0.0541	1.4705	0.0149	0.0417
Breast	0.0045	0.0059	3.6350	0.1474	2.0812	0.0877	0.1525

## Data Availability

The data that support the findings of this study are openly available on GitHub at https://github.com/qczggaoqiang/MST-DC
